# Effect of meal timing on cognitive function: a scoping review

**DOI:** 10.1002/alz70858_107426

**Published:** 2025-12-25

**Authors:** Linlin Ding, Meina Zhang, Chooza Moon

**Affiliations:** ^1^ The University of Iowa, Iowa City, IA, USA

## Abstract

**Background:**

Disruptions in circadian rhythms may increase the risk of developing and worsening Alzheimer's disease and related dementias (ADRD). Meal timing is one of the zeitgebers that helps synchronize the circadian rhythms. Recent studies indicate that meal timing patterns may influence cognitive function. However, there is still debate regarding whether specific types of meal timing are beneficial for improving cognition. This review aims to explore the potential associations between distinct meal timing patterns and cognitive function.

**Method:**

The search was conducted using electronic databases such as PubMed, CINAHL, PsycINFO, and Scopus. Initially, 2, 896 studies were identified. After applying inclusion and exclusion criteria, 12 studies met the eligibility criteria for the review.

**Result:**

The studies analyzed included populations of varying sample sizes, with the number of participants ranging from 26 to 3,111, aged 15.9 to over 60 years old. This review presents evidence suggesting that most studies focused on Time‐Restricted Feeding (TRF), Intermittent Fasting (IF), consuming meals earlier in the day may enhance cognitive function. Some studies indicate specific meal timings can influence cognitive function differently. Eating lunch after 12:00 PM may enhance cognitive performance, while consuming meals during night shifts can have a negative effect. However, only one study reported an association between TRF and a higher prevalence of cognitive impairment (CI) among older adults.

**Conclusion:**

The findings of this review indicate that association between meal timing and cognitive function. However, most studies reviewed were cross‐sectional with small sample sizes and did not account for comprehensive covariates related to circadian rhythms, such as sleep patterns and activity levels. Future research should prioritize longitudinal and interventional studies with larger, more diverse samples to better understand the relationship between meal timing and cognitive function.

